# Metagenomics and Culture Dependent Insights into the Distribution of Firmicutes across Two Different Sample Types Located in the Black Hills Region of South Dakota, USA

**DOI:** 10.3390/microorganisms9010113

**Published:** 2021-01-06

**Authors:** Tanvi Govil, Manasi Paste, Dipayan Samanta, Aditi David, Kian Mau Goh, Xiangkai Li, David R. Salem, Rajesh K. Sani

**Affiliations:** 1 Department of Chemical and Biological Engineering, South Dakota School of Mines and Technology, Rapid City, SD 57701, USA; tanvi.govil@mines.sdsmt.edu (T.G.); dipayan.samanta@mines.sdsmt.edu (D.S.); Aditi.David@mines.sdsmt.edu (A.D.); 2 Composite and Nanocomposite Advanced Manufacturing—Biomaterials Center, Rapid City, SD 57701, USA; 3 Department of Computer Science and Engineering, South Dakota School of Mines and Technology, Rapid City, SD 57701, USA; Manasi.Paste@mines.sdsmt.edu; 4 Faculty of Science, Universiti Teknologi Malaysia, Johor 81310, Malaysia; gohkianmau@utm.my; 5 MOE, Key Laboratory of Cell Activities and Stress Adaptations, School of Life Science, Lanzhou University, Lanzhou 730000, China; xkli@lzu.edu.cn; 6 Department of Materials and Metallurgical Engineering, South Dakota School of Mines and Technology, Rapid City, SD 57701, USA; 7 BuG ReMeDEE Consortium, Rapid City, SD 57701, USA

**Keywords:** landfill compost, diversity, Firmicutes, *Geobacillus*, metagenome, SURF

## Abstract

Firmicutes is almost a ubiquitous phylum. Several genera of this group, for instance, *Geobacillus*, are recognized for decomposing plant organic matter and for producing thermostable ligninolytic enzymes. Amplicon sequencing was used in this study to determine the prevalence and genetic diversity of the Firmicutes in two distinctly related environmental samples—South Dakota Landfill Compost (SDLC, 60 °C), and Sanford Underground Research Facility sediments (SURF, 45 °C). Although distinct microbial community compositions were observed, there was a dominance of Firmicutes in both the SDLC and SURF samples, followed by Proteobacteria. The abundant classes of bacteria in the SDLC site, within the phylum Firmicutes, were Bacilli (83.2%), and Clostridia (2.9%). In comparison, the sample from the SURF mine was dominated by the Clostridia (45.8%) and then Bacilli (20.1%). Within the class Bacilli, the SDLC sample had more diversity (a total of 11 genera with more than 1% operational taxonomic unit, OTU). On the other hand, SURF samples had just three genera, about 1% of the total population: *Bacilli*, *Paenibacillus*, and *Solibacillus.* With specific regard to *Geobacillus*, it was found to be present at a level of 0.07% and 2.5% in SURF and SDLC, respectively. Subsequently, culture isolations of endospore-forming *Firmicutes* members from these samples led to the isolation of a total of 117 isolates. According to colony morphologies, and identification based upon 16S rRNA and gyrB gene sequence analysis, we obtained 58 taxonomically distinct strains. Depending on the similarity indexes, a gyrB sequence comparison appeared more useful than 16S rRNA sequence analysis for inferring intra- and some intergeneric relationships between the isolates.

## 1. Introduction

Phylum Firmicutes is widespread in nature. Many of its members are spore-forming Gram-positive bacteria and are an essential part of the microbial community associated with lignocellulosic biomass degradation and carbohydrate polymers decomposition. Therefore, Firmicutes are of importance when ligninolytic bacteria and enzymes are desired [1]. Firmicutes comprise classes Bacilli, Clostridia, Erysipelotrichia, Limnochordia, Negativicutes, Thermolithobacteria, and Tissierellia. Both Clostridia and Bacilli are more well studied and the members of Clostridia are typically anaerobic fermenters, while the members of Bacilli are facultative aerobes which have given them a unique ability to replicate rapidly across adverse environmental conditions [2]. Many Bacilli-like bacteria are demonstrated as beneficial microbes widely used in industry and agriculture [3,4,5]. One of its member genera, the *Geobacillus,* is the predominant contributors to the hydrolytic degradation of organic matter when thermophilic conditions persist [6].

To better understand microbial diversity, biogeographical distribution, and the functions of members of Firmicutes in soils, an increasing number of studies have tried to establish the linkage between microbial community composition/diversity and their locations. For example, diversity studies have revealed the apparent domination of the members of this class in geothermal sources with temperature optimums ranging from 45 to 70 °C, such as hot springs [7,8,9,10,11,12] and deep-sea hydrothermal vents [13,14,15,16,17,18,19] to name a few. The thermophilic family *Bacillaceae* also account for the most prevalent group of thermophiles present during the thermophilic temperature phases in the compost, with represented genera of *Geobacillus, Bacillus, Ureibacillus, Brevibacillus, Paenibacillus,* and *Aneureinibacillus*, now highlighted in many studies [20,21,22,23,24,25]. In contrast, Larkin et al. (1966) reported the distribution and diversity of ninety psychrophilic isolates of *Bacillus* from the soil, mud, and water by selective enrichment at 0 °C [26]. Also, psychrophilic and psychrotolerant *Bacillus marinus* strains were isolated from tropical Atlantic, Arctic, and Antarctic deep-sea sediments, and authors reported that some of the isolates are even extremely psychrophilic, having maximum growth temperatures of 4 °C [27]. Therefore, it is not surprising that numerous *Bacillus* strains capable of growing at low temperatures could be isolated from many different habitats [28,29], where the strains use a variety of mechanisms that help in cold acclimation [30,31,32]. Interestingly, thermophilic *Geobacillus* members, such as *Geobacillus debilis*, have been isolated from a cool soil environment in Northern Ireland [33], where the cells were found to be existing not only as endospores but also as vegetative cells.

In fact, the members of *Geobacillus* are so prolific in the diversification of their genomes via horizontal gene transfer, inductive mutation, and transposable elements that, since the time they were classified as a separate genus derived from *Bacillus* spp. in 2001, there has been a tremendous expansion of the number of species in *Geobacillus* that have been isolated from diverse environments and sequenced. To date, the total number of genome sequencing projects involving *Geobacillus* is 126, with GenBank IDs publicly available for 89 of those projects with the GOLD database of Joint Genome Institute (https://gold.jgi.doe.gov/project?id=Gp0005586). Based on the whole genome sequences, the database mentions the names of 17 representative species within the genus: *G. thermodenitrificans, G. stearothermophilus, G. thermoleovorans, G. thermocatenulatus, G. kaustophilus, G. zalihae, G. caloxylosilyticus, G. gargensis, G. toebii, G. proteiniphilus, G. uzenensis, G. yumthangensis, G. lituanicus, G. icigianus, G. vulcani, G. jurassicus,* and *G. subterraneus*, as well as the classification of many of the strains that have not been assigned to named species. However, by 16S rRNA gene sequence analysis, these 17 species have been clustered into seven closely related clades, represented by *G. kaustophilus, G. thermodenitrificans, G. stereothermophilus, G. thermoleovorans, G. caldoxylosilyticus, G. thermocatenulatus,* or *G. thermoglucosidasius* [6,34,35,36]. Within the *Geobacillus* species, the sequence similarity of the 16S rRNA is >97% [37], thus highlighting the redundancy of using 16S rRNA gene sequence analysis alone for the taxonomic classification of *Geobacillus* species at a genus, species, or subspecies level.

As an alternative to the 16S rRNA gene, the utilization of conserved protein-coding genes, such as recN [6,34,35,36], recA, and rpoB [38], has been reported for *Geobacillus* species resolution. The gyrB gene that encodes the subunit B protein of a type II DNA gyrase topoisomerase and is distributed universally among bacterial species has been used in the phylogenetic studies of *Bacillus, Pseudomonas, Acinetobacter, Mycobacterium, Aeromonas*, as well as the *Salmonella, Shigella,* and *Escherichia coli* groups [39].

In the present study, we set out to accomplish two goals. First, we present the results of diversity detected with the help of 16S rRNA high throughput metagenomics (HTM) within the Firmicutes phylum isolated from two of the sites: South Dakota Landfill Compost (SDLC, 60 °C) and Sanford Underground Research Facility sediments (SURF, 45 °C). From this work, we present a case that Firmicutes are generalists for habitat and substrate and are one of the important components of the soil microbial community whose distribution may be unaffected by soil composition or prevailing environmental conditions. Members of the Firmicutes are present in landfill sites with a likely role in landfill cellulose decomposition [40]. In addition, Firmicutes have been shown to reduce sulfate and iron and are considered to play a role in the in-situ bioremediation of impacted soils [41]. Hence, finding Firmicutes with high abundance and diversity in both these sites will provide support for the hypothesis that Firmicutes have multiple roles to play in the environment, with many of their niche functions potentially unexplored.

This study provides the first descriptions of functional diversity of landfill biomass-degrading communities, demonstrating the significant potential of landfill sites for the provision of novel CAZymes of ecological and biotechnological significance.

Secondly, we compare 16S rRNA gene-based and gyrB-based phylogenies of the thermophilic members, including *Geobacillus* species isolated from these samples, and evaluate the utility of gyrB as an alternative to 16S rRNA gene sequencing to precisely resolve relationships among sequenced strains within this group. In sum, this study assesses a combined use of culture-dependent and culture-independent metagenomics to analyze the diversity and distribution patterns of Firmicutes across two sampling sites located in the Black Hills region of South Dakota, USA.

## 2. Materials and Methods

### 2.1. Collection of Samples

In the July of 2017, samples were collected from an aerobic landfill and treatment center (Rapid city Solid Waste in State of South Dakota, USA, 44°01′56″ N 103°11′59″ W) and used as sources for the isolation of thermophilic bacteria. Four samples were collected from a self-heating yard waste/municipal solid waste (MSW) compost pile (where yard waste and MSW is in the ratio of 50:50), at a distance of approximately 50–70 cm inside the hot pile (55–60 °C), which were then mixed to form one sample (referred to as SDLC sample in the manuscript). At the time of sampling, the compost piles were sufficiently dry to be archived, and the physiochemical properties are listed in Table 1. The compost samples obtained were collected in a 1-gallon sealable plastic bag and were then immediately transferred to the laboratory in a Styrofoam container with ice.

The second sample used in this study for analyzing the microbial diversity was a composite sample collected in July 2018 from Sanford Underground Research Facility (SURF) in Lead, South Dakota, USA (44°21′17″ N 103°45′09″ W), from the 4850 ft depth level Ross campus that includes the Ross Shaft and #6 Winze. Roughly eight water, mat, and adjacent sediment sub-samples were collected from the Ross shaft with sampling sites within a radius of 100 m, and at different depths (0–3 mm, 3–6 mm, and 6–9 mm), in order to obtain a horizontal as well as a vertical profile of the site, which were then mixed to form a composite sample (referred to as SURF sample in the manuscript). The method of the sampling is provided in our previous studies [42], and the temperature and pH were determined in situ using a portable combined tester that measures pH, conductivity, salinity, dissolved oxygen (DO), total dissolved solids (TDS), and temperature (HQ30D53000000, Hach). Samples for analysis of organic matter, organic carbon, total nitrogen, ammonia, and C/N ratio were sent to UC Davis, CA, USA. Both the samples were stored at −20 °C for subsequent DNA isolation and sequencing analysis.

### 2.2. DNA Extraction, 16S rRNA Gene Amplification, and Sequencing for Metagenomic Analysis

The total genomic DNA from the SDLC and the SURF sample was extracted following the MINES protocol described elsewhere [43]. After checking the DNA’s quality using a Nano-Drop 2000 Spectrophotometer (Thermo Fisher Scientific, Waltham, MA, USA), the DNA was stored at −20 °C, until further analysis. The following 16S rRNA gene amplification, library creation, Illumina 2 × 250-bp sequencing, and diversity analysis, were done using the services of RTL genomics (Lubbock, TX, USA). During the procedure, the primer pairs used for analyzing bacterial diversity were 515F (5′GTGCCAGCMGCCGCGGTAA3′) and 806R (5′GGACTACHVGGGTWTCTAAT3′) [44]. For the analysis of archaeal diversity, the primers used were 517F (5′GCYTAAAGSRNCCGTAGC3′) and 909R (5′TTTCAGYCTTGCGRCCGTAC3′) [45]. The metagenomic data from this study has been deposited in the U.S. National Center for Biotechnology Information and is available through accession number PRJNA684582.

### 2.3. Metagenomics Sequencing Data Analysis

The sequence analysis of the raw reads obtained from the Illumina sequencer was performed using the RTL Genomics analysis pipeline [46]. Briefly, the steps included the merging of the paired ended reads, performing quality trimming, removing chimera sequences, performing clustering at a 4% divergence using the USEARCH clustering algorithm, classifying the clusters into operational taxonomic units (OTUs) based on an open reference clustering approach using the UPARSE OTU selection algorithm [47], estimating the abundance of each OTU in the population, and taxonomic assignment using a high-quality sequences database derived from National Center for Biotechnology Information (NCBI). The diversity analysis was next performed using the RTL The genomics diversity analysis program, phyloseq (Version 1.14.0) [48] finally retrieved the output in the form of both the trimmed and full taxonomic information for each level, where the level is Kingdom, Phylum, Class, Order, Genus, or Species. It is important to note that the data are not strictly quantitative, and thus all percentages reported should be taken as relative abundance only. The relative abundance of individual taxa within each community was estimated by comparing the number of sequences assigned to a specific taxon against the number of total sequences obtained for that sample and expressing as a percentage. The Shannon–Weaver index as an index of Alpha diversity at the species level was calculated using the Al Young studios online tool (https://www.alyoung.com/labs/results.html). The Sørensen pairwise dissimilarity as a measure of Beta-diversity was calculated and analyzed using betapart_v1.5.2 [49]. Statistical significance between two samples was analyzed using Student’s t-test by SPSS 22.0 software (IBM, Armonk, NY, USA), and differences were considered significant when *p* < 0.05.

### 2.4. Enrichment and Isolation

Culture isolations for aerobic endospore forming Firmicutes members were performed by enriching the SDLC and SURF samples following the strategies of nutritional and physical constraints devised by Priest et al. (1989). Roughly 1 g of the samples were weighed, and while some samples were pasteurized at 80 °C for 10 min, another set of samples was subjected to 50% ethanol treatment for 30 min to eradicate vegetative cells. Next, while the SDLC enriched samples were inoculated into 100 mL of the mineral base salt solution (MBSS) medium (pH 8.0) containing 0.25 g potassium nitrate, 0.1 g monopotassium phosphate, 0.1 g magnesium sulphate, 0.1 g yeast extract, 0.005 g sodium molybdate, and 0.02 g calcium chloride, with 0.5% corn stover as the sole carbon source, and incubated at 60 °C for three days. The SURF sample was inoculated into 100 mL of the MBSS with identical composition and carbon source, but with a pH at 6.0 and temperature at 45 °C. After the required incubation, the enriched samples (designated as the 100% stock solution) were diluted by a factor of ten in 9 mL of sterilized saline solution (0.9% NaCl). Finally, 0.1 mL from each dilution tube was plated on MBSS plates supplemented with 2% agar and glucose as the carbon source, using spread plating. The plates were incubated for 24 h at 60 °C and 45 °C for SDLC and SURF samples, respectively. Cultures showing different colony morphology were picked and purified by streaking onto the same medium at least three times. The purity was confirmed after microscopic observation of a single morphological type per culture. All of the isolates were routinely maintained at 4 °C on Luria Broth (LB) agar slants and stored at −80 °C in LB broth cultures supplemented with 80% glycerol 1:1 ratio. Isolates were designated according to their geothermal area of origin, the sample number taken from that origin, and the number of the isolates obtained in that sample.

### 2.5. Amplification of 16S rRNA and gyrB from the Isolates Using Colony PCR

While 16S rRNA from the isolates was amplified using primer pairs 505F (5′-GTGCCAGCMGCCGCGGTAA-3′) and 806R (5′-GGACTACHVGGGTWTCTAAT-3′) [50], for amplifying gyrB, the primer pairs used were gyrF (5′-GAAGTCATCATGACCGTTCTGCATCGCTCAGGGTCAGGGTCAGAAAGTTTCGA-3′) and gyrR (5′-AGCAGGGTACGGATGTGCGAGCCAGTCTCAGACAGTCTCAGGCAGTCTCAGGTAT-3′) [51]. For both the reactions types, for each of the tested bacteria, the PCR mixtures (25 μL) used for amplification contained 12.5 uL of 2X Green GoTaq master mix (Promega: contains dNTPs, MgCl_2_, and Taq polymerase), 2.5 µL of 10 µM forward primer, 2.5 µL of 10 µM reverse primer, and 7.5 µL of nuclease free water (Promega), mixed in a 0.2 mL microcentrifuge tube. For colony PCR, each bacterium was added to the appropriate PCR tube by taking a micropipette tip, poking a colony, and sloshing it into the reaction mixture. The control contained no bacteria. PCR amplifications were completed by setting each of the reaction tubes in a BIO-RAD T100 thermal cycler, using the following cycling parameters: For 16S gene amplifications: A: Initial denaturation at 95 °C for 2 min, B1: Denaturation at 95 °C for 40 s, B2: Annealing at 55 °C for 1.5 min, B3: Extension at 72 °C for 1.5 min, B4: Repeat Step B1-B3 for 35 times, C: Final extension at 72 °C for 2 min, and D: Hold at 4 °C until used for analysis. For gyrB amplification: A: Initial denaturation at 95 °C for 3 min, B1: Denaturation at 94 °C for 1 min, B2: Annealing at 60 °C for 1 min, B3: Extension at 72 °C for 1.5 min, B4: Repeat Step B1–B3 for 35 times, C: Final extension at 72 °C for 2 min, and D: Hold at 4 °C until used for analysis. PCR products were viewed under UV light after standard ethidium bromide gel electrophoresis. PCR products were purified with GenElute™ PCR Cleanup Kit (Sigma). The sequencing of bacterial 16S rDNA and gyrB amplicons was done by Molecular Cloning Laboratories (MCLAB, South San Francisco, CA, USA) using the primers, as mentioned earlier.

### 2.6. Identification of the Isolates

Raw data of DNA sequences were analyzed with Flinch TV. A nucleotide BLAST search with NCBI was performed in order to obtain information on the phylogenetically closest relative. Multiple sequences alignment was performed using the program MEGA7 [52]. Phylogenetic trees were constructed by the neighbor-joining method with bootstrap values based on 1000 replications in MEGA7. Evolutionary distances were calculated by Kimura’s two-parameter model. Moreover, Gram staining for each of the isolate was carried out following the methods of Coico as described previously [53].

## 3. Results and Discussion

### 3.1. Bacterial Community Analysis

In this study, we used amplicon sequencing to investigate the microbial distribution of endospore forming Firmicutes in two different sampling areas across Black Hills, SD, USA. Based on the identified 16S rRNA gene reads from the SDLC and SURF sediment metagenome, the total OTUs (Archaea and Bacteria) in SDLC and SURF were 66,738, and 106,144, respectively. This indicates that the total number of cells decreased as the soil temperature increased from 35–45 °C in the SURF sample to 55–65 °C in the SDLC sample. Thus, the difference in temperature could be one of the reasons for a significant difference in OTUs of both sites (*p* = 0.007).

**Figure 1:**
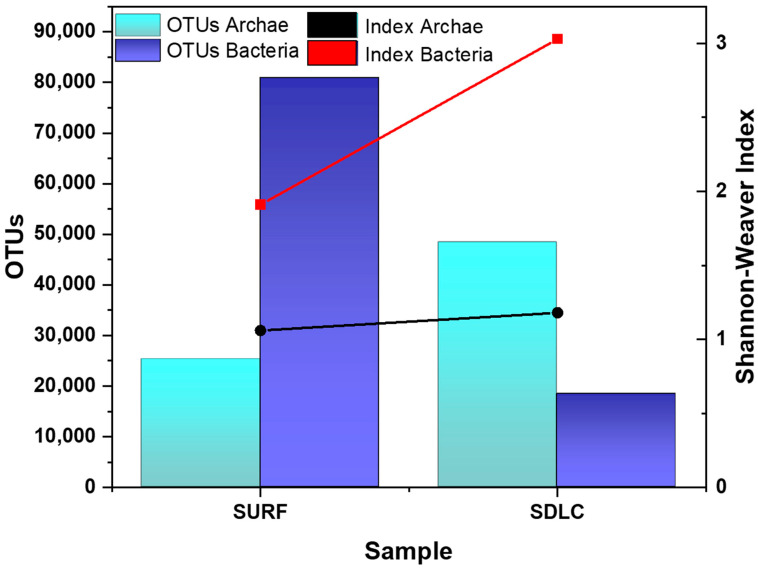
Sample type vs. total species diversity plot as indicated by number of OTUs (Left Y axis) and Shannon-Weaver index (Right Y axis). Here, SURF is Sanford Underground Research Facility (45 °C), SDLC is South Dakota Landfill Compost (60 °C), OTU is Operational taxonomic unit (OTU).

In the SURF sample, the microbial communities were dominated by Bacteria with OTUs of 80,885 (Archaea was 25,259), which contributed approximately 73.2% of the total number of cells. However, in the 60 °C SDLC sample, Archaea became the dominant population with total OTUs of 48,367 (Bacteria was 18,371), and it accounted for more than 72.4% of the total number of cells. Moreover, between them, higher alpha diversity was observed in the SDLC (Shannon–Weaver index: Bacteria, 3.03; Archaea, 1.18), than in the SURF samples (Shannon–Weaver index: Bacteria, 1.91; Archaea, 1.06) (Figure 1). This indicates that the higher temperature SDLC’s bacterial community profiles were substantially more complex than those of the lower temperature SURF site, with the archaeal diversity profoundly the same across both the sites. It is apparent in the literature that temperature, available organic matter, and C/N ratio are the key factors in the selection of microbial communities [54]. In the SDLC compost, the levels of organic carbon, total carbon, and C/N ratios were higher than in the SURF samples (Table 1). Further, in the self-heating compost compiles, there are a lot of organic matter, proteins, and other nutrients such as nitrogen and phosphorus, which support soil biological activity and functional diversity [54]. Thus, the higher alpha diversity for the OTUs obtained for the metagenome of the SDLC compost could be due to the increase in complexity of the thermophilic communities with increases in levels of some physicochemical parameters like temperature, DO, water content, total organic matter, and total carbon in SDLC as compared to SURF.

Contrastingly, the unique deep subsurface of this mine is characterized by the presence of different water-soluble ions (sulfate, nitrate, iron) in soils [49], with limited amounts of organic matter and total carbon. The SURF site is apparently the deepest mine (2.4 km deep) in the North America and had the largest gold deposit ever found in the Western Hemisphere [55]. Today, the SURF site houses a world-renowned neutrino research lab, with geochemical characterization of soils revealing high amounts of toxic metals such as As, Cd, Co, Cr, Cu, Ni, Pb, Zn, and U [55]. The presence of high metals, especially iron, indicates that the SURF sample where the organic energy sources are limited, can still support the growth of various chemotrophic microorganisms. However, the presence of low organic matter in the SURF site compared to the SDLC site can probably explain why the alpha diversity of bacterial communities in the in the SDLC samples was more enriched compared to the SURF samples.

Further, considering the fact that physiochemical properties in both the locations were very different from each other, we analyzed the samples for their β-Diversity. The Sorenson β-diversity dissimilarity index between the two samples at the phyla, class, and lower taxa was 0.101, 0.27, and 0.403, indicating the two communities were quite similar in sharing the common phyla, but dissimilar in terms of sharing the lower taxa. They also possessed significantly different community compositions. The community composition of phyla in both the SURF and SDLC samples is shown in Table 2. In the whole community of SDLC, the three most abundant phyla were Firmicutes, Proteobacteria (γ-proteobacteria), and Actinobacteria, with relative abundances of approximately 86%, 6% and 6%, respectively. Within the whole SURF community, Firmicutes was still the dominant phylum at 69.5%, but the community was also significantly represented by Proteobacteria (α-proteobacteria and β-proteobacteria) and Bacteroidetes, which had a relative abundance of approximately 5%, and 2%, respectively. In addition, the SURF community had about 16% unclassified bacteria and 4% of its population with no hit.

Out of these, the members of Firmicutes, and Actinobacteria phyla are spore formers, and their combined higher total in SDLC (92%) than in SURF (71.5%) can be assigned to the higher prevailing temperature in SDLC compost (60 °C) over SURF sediments (45 °C). Firmicutes and Actinobacteria form resistant physiological stages that allow them to survive in hostile environments.

Nevertheless, despite so much variation in the two sampling sites in terms of their physiological properties, Firmicutes’ dominance in both the samples is clearly evident. Since the phylum Firmicutes members are endospore-forming aerobic or facultatively anaerobic bacteria, this property makes the phylum hardy in potentially harsh conditions and renders it a phenotypically and phylogenetically diverse taxon. Endospore formation allows these bacteria to be distributed into most of the habitats on Earth. While high-temperature conditions in the SDLC site can be attributed as a prime reason for the abundance of Firmicutes in the SDLC sample, the low carbon and moisture levels can explain their easy outgrowth against other phyla in the SURF subsurface environment.

Further, many of the members of Firmicutes are lignocellulolytic microorganisms as they can degrade tough lignocellulose in the plant biomass via the secretion of lignocellulases like cellulases, hemicellulases, and ligninolytic enzymes. Therefore, the abundance of Firmicutes in the SDLC sample rich in municipal solid wastes and yard waste is an anticipated event. Members of the Firmicutes phylum associated with biomass conversion are consistently abundant in both culture-based and 16S rRNA gene inventories of landfill sites, leading to the suggestion that they are the predominant degraders of biomass in landfill [40]. However, the prevalence of Firmicutes in SURF samples with low organic matter content is a bit surprising. In the previous studies dealing with microbial community structures analysis in the subsurface environment, Proteobacteria, known to have sulfate reducers, nitrate reducers, and chemolithotrophic metabolizers, had been the dominant phyla in the majority of them [55,56]. However, the dominance of Firmicutes over Proteobacteria in this study, unaffected by sampling conditions having high metal concentrations, indicates a potential niche that remains unexplored for the members of Firmicutes. Gupta et al. 2018 demonstrated the ability of Firmicutes members to reduce sulfate and iron and presented a case for the role of Firmicutes for the in-situ bioremediation of an acid mine drainage impacted soil [41]. Similar revelations have been made by authors of other studies [57]. A large suit of metal resistance genes in the bacterial phyla Firmicutes was recently reported by Yong et al. (2018) [58]. Therefore, ecologically, prevalence of Firmicutes in the Black soils of South Dakota signifies that this region represents a repository of biomass-degrading diversity that can be a rich source of new enzymes as well as metal resistance genes for environmental bioremediation. Further, these results support our hypothesis that members of Firmicutes are indeed versatile and can often be detected at a relatively high abundance in diverse environments, without strong dependence on soil composition or prevailing environmental conditions.

#### 3.1.1. Community Composition of the Phylum Firmicutes

At the class level in SDLC, the most dominant class was Bacilli at 83%, followed by Clostridia at 3%. By contrast, the SURF sediments revealed a dominance of Clostridia at 46% over Bacilli at 20%. The shift in bacterial dominance from Bacilli to Clostridia in SURF sediments can be due to comparatively low oxygen conditions in this site, with Clostridia members preferring anaerobic conditions.

**Figure 2:**
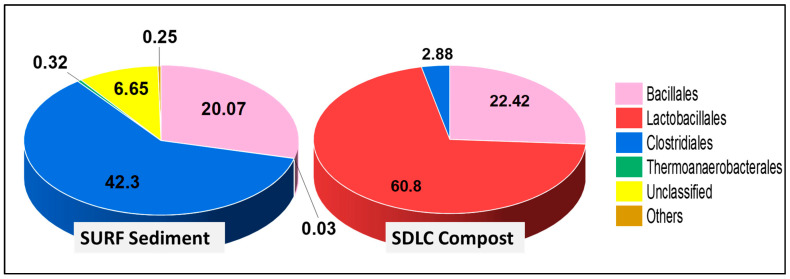
Composition and relative abundance of phylum Firmicutes in SDLC and SURF samples at the Order level. Sequences that could not be classified into any known group were assigned as “unclassified”. Orders making up less than 0.1% of total composition were classified as “other”. The numbers here indicate percent relative abundance of the mentioned orders.

The dominance of members of Bacilli over Clostridia was evident down the phylogenetic levels in SDLC samples, where the order Lactobacillales (60.8%) was higher than Bacillales (22.4%) and Clostridiales (2.9%) (Figure 2). Further, while the order Bacillales in SDLC was represented by a total of five families (Bacillaceae, Planococcaceae, Staphylococcaceae, Paenibacillaceae, and Thermoactinomycetaceae at 13%, 7.8%, 0.61%, 0.60%, and 0.29% respectively), Lactobacillales had a total representation from six families (*Aerococcaceae,* Lactobacillaceae, Enterococcaceae, Carnobacteriaceae, Leuconostocaceae, and also Streptococcaceae at 21%, 16%, 13%, 6.1%, 4.5%, and 0.04%, respectively). By contrast, only two families represented Clostridiales (Clostridiaceae at 2.1%, and Eubacteriaceae at 0.78%). The prevalence of Lactobacillales over other orders in the SDLC sample can be correlated to the acidic pH conditions of the sample. Members of Lactobacillales are responsible for breaking down the organic waste materials into organic acids with an accompanied drop in pH in the compost [59]. While the organic matter is being transformed, heat is released during the process, favoring actinomycetes and thermophilic bacteria such as *Bacillus* sp. This explains the presence of Bacillales as the second most abundant order in the SDLC sample.

In comparison, within the SURF sample at the order level, there were no Lactobacillales at all, and Clostridiales (42%) still were prominent, with Bacillales at 20%. Here, there were only three prominent families within Bacillales represented by Bacillaceae, Planococcaceae, and Paenibacillaceae at 11%, 7.9% and 2.9%, respectively. However, the diversity of classes presented in SURF samples composing Clostridiales was relatively high, with a total of six families >0.1%, being Clostridiaceae (24%), Symbiobacteriaceae (7.6%), Ruminococcaceae (7.6%), Lachnospiraceae (1.2%), Peptococcaceae (0.34%), and Gracilibacteraceae (0.19%). This again highlights that while the diversity within the Bacillilales and Lactobacillales group was higher in the 60 °C SDLC samples, the 45 °C SURF samples had a higher diversity of Clostridiales and Thermoanaerobacterales, i.e., the anaerobic groups.

**Figure 3:**
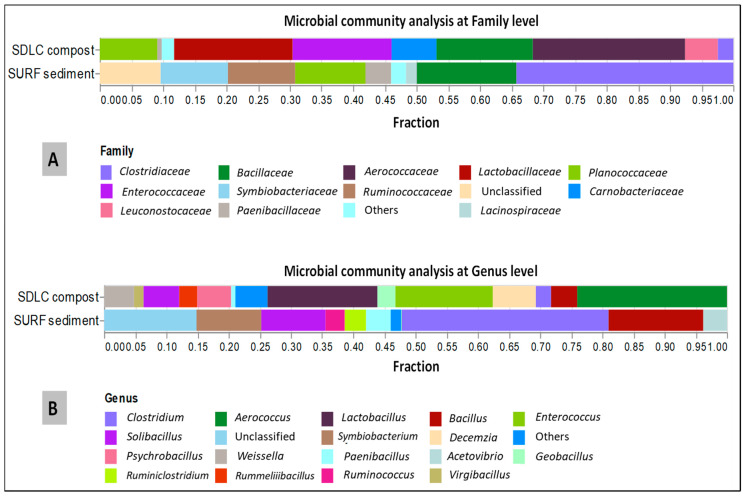
Composition and relative abundance of Firmicutes in SDLC and SURF samples on the (**A**) Family level, (**B**) Genus level. The color of the column represents the different genera, and the length of the column represents the fractional size of the family/genus. Sequences that could not be classified into any known group were assigned as “unclassified”. Genera making up less than 0.1% of total composition in both the samples were classified as “other”.

The leading bacterial communities for each sample was also analyzed at the genus level (Figure 3). In the SDLC samples at the genus level, at least ten genera with >4% abundance were detected. Among them, *Aerococcus, Lactobacillus, Enterococcus, Desemzia, Solibacillus, Psychrobacillus,* and *Weissella* were the six most abundant genera with relative abundances of 21%, 15%, 13%, 5.9%, 4.9%, 4.7%, and 4.1%, respectively. *Bacillus, Geobacillus, Rummeliibacillus, Clostridium*, and *Virgibacillus* were the next five dominant genera with 3.6%, 2.5%, 2.5%, 2.1%, and 1.3%, respectively. In SDLC, *Lactobacillus, Paenibacillus, Leuconostoc, Gracilibacillus, Staphylococcus, Planococcus, Garciella*, and *Planifilum* were the minor genera detected with much lower average relative abundances (less than 1% across SDLC). By comparison, in the SURF samples, while the *Clostridium, Bacillus, Symbiobacterium,* and *Solibacillus* at 24%, 11%, 7.6%, and 7. 6%, were the three most abundant genera (more than 5%), the presence of *Paenibacillus* (2.9%), *Acetivibrio* (2.78%), *Ruminiclostridium* (2.51%), *Ruminococcus* (2.25%) were also detected in the sediments with more than 1% relative abundance.

In the SDLC site, the class Bacilli had a clear dominance and more diversity (11 genera with more than 1% OUTs) than in the SURF site, which had just two genera above 1% in the class Bacilli: *Paenibacillus*, and *Solibacillus.* For Clostridia in the SDLC sample (present at only 2.9%), only one genus, *Clostridium,* was above 1%, but the diversity was higher in the SURF sample, where Clostridia (present at 46%) had five genera: *Clostridium, Acetovibrio, Ruminiclostridium, Ruminococcus,* and *Symbiobacterium.* Another feature of the SURF samples was the presence of unclassified family at 10% of the OTUs, and 3.5% of the OTUs being unclassclassified at the phylogenetic class level and lower levels. This comparison of the phylogenetic diversity at the level of genera between the samples highlights, again, the presence of higher bacterial diversity in the SDLC compost, as reflected in the large difference in the Shannon–Weaver index between the SDLC and SURF samples. Moreover, the abundance of Bacilli in the SDLC compost of this study, matches the results in some other studies, where it was mentioned that some members of the Bacillus genus have the ability to assimilate nitrogen and reduce the ammonium nitrogen loss during composting [60].

#### 3.1.2. Community Composition of the Phylum Actinobacteria

**Figure 4:**
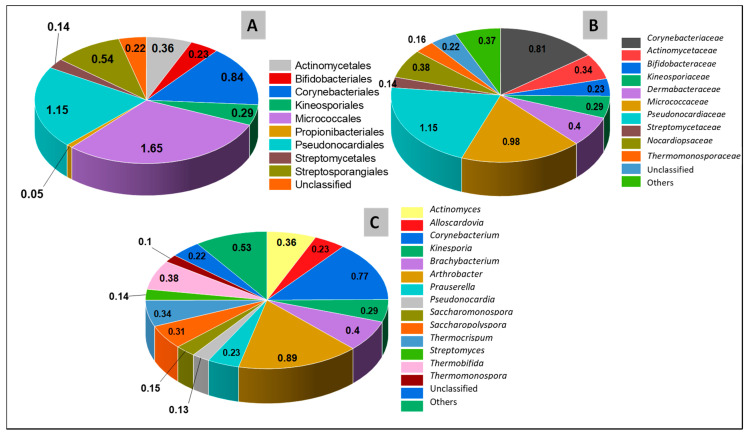
Composition and relative abundance of Actinobacteria in SDLC sample on the (**A**) Order level, (**B**) Family level, (**C**) Genus level. The color of the pie represents the different genera, and the numbers represents the percentage of the total composition at each level. Sequences that could not be classified into any known group were assigned as “unclassified”. Genera making up less than 0.1% of total composition in both the samples were classified as “other”.

Beyond Firmicutes, the second phylum that is known to be spore producers and was present in the SDLC samples was Actinobacteria at 5.5% (Figure 4). These actinobacteria belonged to a single class of Actinobacteria, subdivided into nine orders (>0.1% relative abundance), ten families (>0.1% relative abundance), and fourteen genera (>0.1% relative abundance). There was no presence of this phylum in the SURF sample, and this difference probably indicates the natural capacity of compost sites to enrich endospore-forming communities. Actinobacteria are bacteria with slow growth rates, but which have a greater capacity to degrade less biodegradable, complex organic compounds compared to other bacteria. They are often distributed in compost sites, whereby they can decompose complex mixtures of polymers in dead plants and animals, and make the carbon available to other prevailing communities [59,61].

#### 3.1.3. Community Composition of Other Bacterial Phyla

Proteobacteria made up the second most dominant phylum in both the samples, with SDLC samples having 6.6% of Proteobacteria, and SURF sample having 5.4% of this phylum. Comparatively higher dominance of phylum Proteobacteria in the SURF site corroborated previous reports, as lineages of Proteobacteria are well-known to survive in low-nutrient environments, metal reduction, and metal resistance [55]. Within this phylum, SDLC samples showed a dominance of γ-proteobacteria at 5.5% > β-proteobacteria (0.81%) > α-proteobacteria (0.33%). Within γ-proteobacteria, the dominant orders were represented by Pseudomonadales (3.4%) and Enterobacteriales (1.8%), and the three most represented families with more than 1% relative abundance were Moraxellaceae (2.0%), Enterobacteriaceae (1.8%), and Pseudomonadaceae (1.4%). At the genus level, the three most dominant genera having a representation of >1% were Acinetobacter (2.0%), Pseudomonas (1.4%), and Enterobacter (1.4%).

SURF samples showed a predominance of α-proteobacteria (4.1%) > β-proteobacteria (1.1%) > δ-proteobacteria (0.20%). All of these classes were solely represented by a single genus, order and family, i.e., α-proteobacteria by *Rhodobacter* (Order, Rhodobacterales; Family, Rhodobacteraceae) at 4.1%, β-proteobacteria by *Alcaligenes* (Order, Burkholderiales; Family, Alcaligenaceae) (1.14%), and γ-proteobacteria majorly by *Pseudomonas* (Order, Pseudomonadales; Family, Pseudomonadaceae) (0.2%). The analysis of the Shannon–Weaver index for species diversity of phylum Proteobacteria in SURF sediments of 0.64 vs. in that of SDLC of 2.21, again indicates the SDLC compost to be much more diverse in its representation across all the phyla.

Bacteroides was also present in both the samples, but while it was 0.56% in SDLC, the population was 1.9% in SURF. Beyond these phyla, SDLC samples just had Deinococcus-Thermus at 0.10%, with the rest of the population unclassified at 0.53%, and “No hits” at 0.61%. By comparison, SURF samples also had Cloroflexi (0.88%), Acidobacteria (0.65%), Spirochaetes (0.44%), Planctomycetes (0.37%), as some more of the representative phylum’s, with the unclassified phyla at 16.3%, and “No hits” being 4.3%. The presence of such a high a population of unclassified and no hits increases the probability of isolating novel species from the SURF site.

Since the primary aim of this work was to investigate the distribution of bacterial communities, in particular Firmicutes in the studied sites, we have not included in-depth discussion on distribution of arachael diversity in this paper. Howsoever, it may be noted that the archaea communities at both the sites exhibited lower diversity and were most closely affiliated to Euryarchaeota with 48.1%, and 57.6% of the reads in SDLC and SURF samples, respectively. The rest of the community was categorized as unclassified. Euryarchaeota is a diverse group which includes all members of the methanogens and certain thermophiles [62]. While prevalence of methanogens in the SURF site has been reported previously by Rastogi et al. (2009) [55], thermophilic methane oxidation has also been a reported activity within compost heaps [63,64]. Further investigation of the data and analysis of the distribution of archael thermophiles can be a rich source for additional investigations of thermophilic composting microbiology.

### 3.2. Identification of the Culturable Isolates

Since both the samples had Firmicutes as the dominant phyla, samples collected from both the sites were analyzed to evaluate the total thermophilic aerobic bacterial abundance. A total of 117 isolates with different colony morphologies were obtained from the enriched samples from the two sites (77 isolates from SDLC samples, numbered, LC1-77; and 40 isolates from SURF samples, SURF1-40). Coincidentally, all the isolated cells appeared Gram-positive, and based on their initial 16S rRNA gene analysis, it was found that there are a total of eight genera in the SDLC samples, with 43 species belonging to *Geobacillus*, 20 species belonging to *Bacillus*, four isolates of *Aneurinibacillus,* three isolates belonging to *Ureibacillus*, three isolates belonging to *Aeribacillus*, two isolates within *Anoxybacillus*, one isolate within *Parageobacillus,* and one species that was recognized as *Thermoactinomyces*. Out of these 77 isolates, 48 isolates were mere replicates of other representative species, and this bought the number of isolates from the SDLC samples that had different identities, as revealed by 16S identification, to 29 (17 *Geobacillus*, 6 *Bacillus*, two *Ureibacillus*, one *Aeribacillus,* one *Parageobacillus,* one *Aneurinibacillus,* one *Anoxybacillus,* and one *Thermoactinomyces*), representing 37.6% of the total isolates (Supplementary Table S1).

On the other hand, in the SURF samples, isolates belonged to six different genera. Out of 40 isolates from SURF, 18 isolates were members of genus *Geobacillus*, 12 isolates were from genus *Bacillus*, four species belonged to *Paenibacillus*, two species were from *Aeribacillus*, one species was from *Ureibacillus*, and three isolates were from genus *Parageobacillus*. In these SURF samples, 26 isolates out of 40, representing 65% of the isolates, appeared different by 16S rRNA analysis (Supplementary Table S2).

A more detailed analysis reveals total percentage of isolates from genus *Geobacillus* were higher in SDLC (58.61%) than SURF (30.70%); *Bacillus* were higher in SURF (34.60%) than SDLC (20.3%); *Parageobacillus* was higher in SURF (11.5%) than SDLC (3.4%); *Aeribacillus* were higher in SURF (7.7%), whereas *Ureibacillus* was equally represented within isolates from SURF and SDLC at 3.8% and 3.4% respectively. Further on, *Paenibacillus* was only present in SURF samples; while *Aneurinibacillus, Anoxybacillus,* and *Thermoactinomyces* species were only isolated from SDLC samples. Interestingly, after performing enrichment of compost and sediments and then performing isolations, members of *Ureibacillus, Aeribacillus, Aneurinibacillus, Anoxybacillus,* and *Parageobacillus* were also isolated. However, these genera were not detected in the metagenomic analysis.

#### 3.2.1. Phylogenetic Relationship between Isolates from SDLC Sample Based on 16S rRNA Gene Sequence Comparison

**Figure 5:**
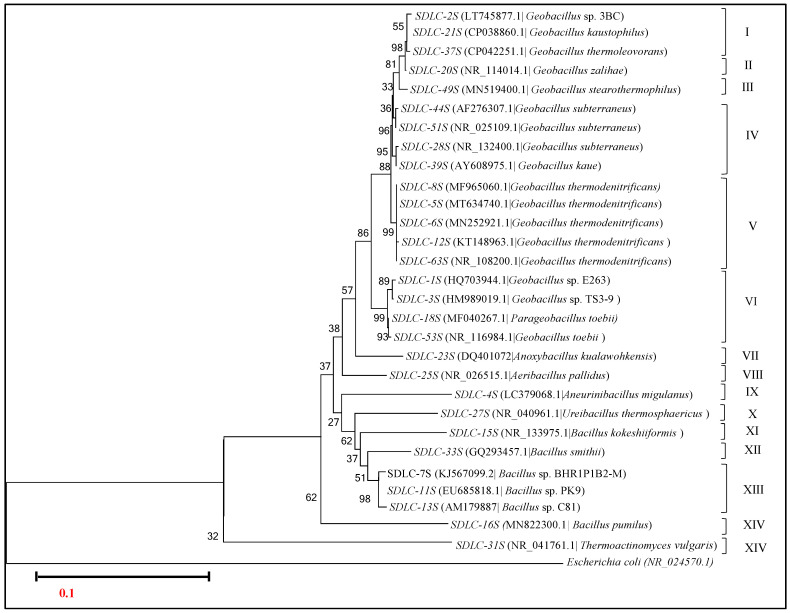
Neighbor-joining phylogenetic tree obtained from 16S rRNA gene sequences of the isolates from the SDLC site. Sequences from this study are shown and isolates closest match from the NCBI database are given in parentheses. The scale bar represents the estimated sequence divergence with an estimated 10 base substitutions per 1000 nucleotide positions. Percentages refer to significant bootstrap values of 1000 calculated trees. *E. coli* was used an outgroup.

**Figure 6:**
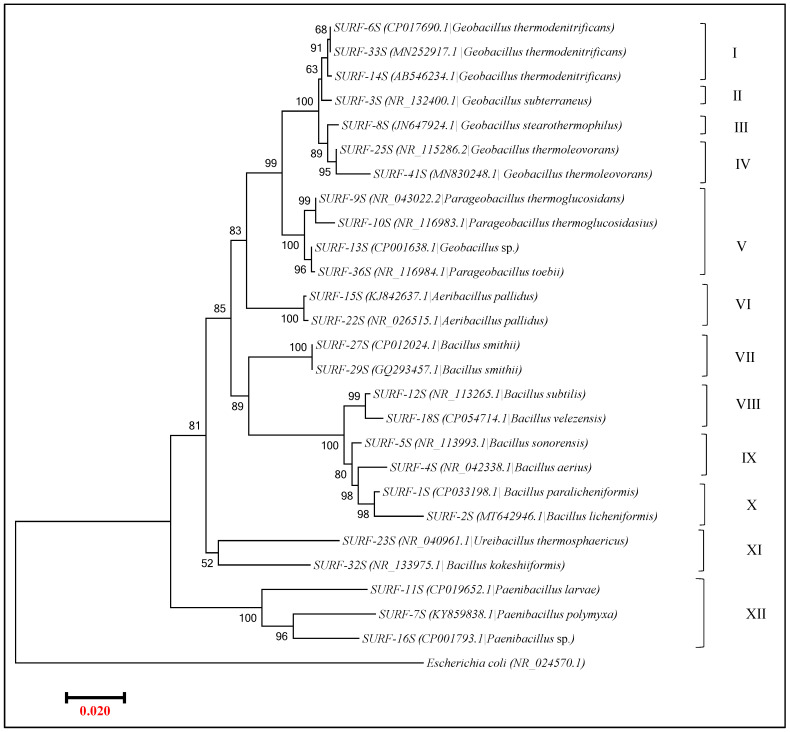
Neighbor-joining phylogenetic tree obtained from 16S rRNA gene sequences of the isolates from the SURF site. Sequences from this study are shown and isolates closest match from the NCBI database are given in parentheses The scale bar represents the estimated sequence divergence with an estimated 20 base substitutions per 1000 nucleotide positions. Percentages refer to significant bootstrap values of 1000 calculated trees.

Next, we compared the 16S rRNA gene sequences of 29 differential isolated strains from SDLC (Figure 5) and 26 of the isolates from SURF (Figure 6). We then checked the similarity index scores amongst their gene sequences using Clustal Omega. According to phylogenetic analysis of the 16S rRNA gene sequences of isolates from the SDLC samples, the isolated strains formed a total of fifteen groups, with ten groups (II, III, VII-XII, XIV and XV) having single species representation (Figure 5). As regards the *Geobacillus* species, based on isolates positioning on the phylogenetic tree, the *Geobacillus* isolates were represented by Groups I-VI. Here, the group I had the isolates representing two different species *G. kaustophilus* and *G. thermoleovorans.* Within group I, there was also an unnamed species strains of *Geobacillus*, numbered 3BC which had 97.61% similarity with *G. kaustophilus,* and 96.87% similarity with *G. thermoleovorans.* Group II and III had single representation with isolates that were identified as *G. zalihae,* and *G. stearothermophilus,* respectively. Group IV had the isolates belonging to clade *G. subterraneus* with a % identity score of 99.16–99.80%*;* group V isolates were identified as *G. thermodenitrificans* with a % identity score of 98.45–99.12%; and group VI supposedly had the isolates found to be close to *G. toebii*. In group VI, an isolate identified as *Parageobacillus toebii* was found to create a sister grouping with *G. toebii,* indicating that although *Geobacillus* and *Parageobacillus* are two ecologically diverse thermophilic genera within the phylum Firmicutes, their isolates are very close to each other phylogenetically and had a similarity score of 99.21%, thus putting a question mark on *Parageobacillus* being classified as a separate genus. Also, group VI had two unnamed species strains of *Geobacillus*, numbered E263, and TS3-9 with a similarity score of 97.20–99.86% between all the four members of the group, as revealed by multiple sequence alignment with Clustal Omega. Overall, the percentage similarity between all the seventeen SDLC isolates belonging to the genus *Geobacillus* was 95.11–99.94%.

Since, from the SDLC samples, we also got the isolates identified to be belonging to different genus: SDLC-23S (as *Anoxybacillus kualawohkensis,* group VII), SDLC-25S (as *Aeribacillus pallidus*, group VIII), SDLC-4S (as *Aneurinibacillus migulanus*, group IX), and SDLC-27S (as *Ureibacillus thermosphaericus*, group X). We also checked the similarity score between these isolates belonging to different genera’s and found that percentage identity between the isolates was 85.65–93.77%. On the phylogenetic tree, while the genus *Anoxybacillus* and *Aeribacillus* were positioned more closely to members of *Geobacillus,* the *Aneurinibacillus*, and *Ureibacillus* isolates were positioned more closely to members of *Bacillus* on the phylogenetic tree (Figure 5). Interestingly, despite being a separate genus, the *Anoxybacillus* isolate SDLC-23S shared 94.10–96.89% identity with other isolates of *Geobacillus*. The *Aeribacillus* isolate SDLC-25S shared 93.31–95.82% identity with members of *Geobacillus* in the SDLC sample. Likewise, SDLC-4S (*Aneurinibacillus* sp.), and SDLC-27S (*Ureibacillus* sp.) had 93.61–95.10%, and 94.80–96.98% identity with the other isolates of *Bacillus.*

Similar observations can be made within the isolates that were identified as *Bacillus* sp. by 16S rRNA analysis (placed in the groups XI-XIV). Herein, group XI had SDLC-15S identified to be *B. kokeshiiformis,* group XII had SDLC-33S identified to be *B. smithii,* SDLC 16S (*B. pumilus*) was present in group XIV, and three unnamed species of (*Bacillus*)—SDLC 7S, 11S and 13S came out to be forming a separate group (XIII). Overall, the percentage similarity between all the six SDLC isolates belonging to the genus *Bacillus* was 88.11–96.94%. Finally, the isolate representing *Thermoactinomycetes vulgaris* was more like an outlier within the isolates, with just 75% on the similarity score with rest of the isolates.

#### 3.2.2. Phylogenetic Relationship between Isolates from SURF Sample Based on 16S rRNA Gene Sequence Comparison

While comparing the 16S rRNA gene sequences of 26 differential isolated strains from SURF, the strains were found to form twelve groups, with group VII-XI representing isolates that resembled *Bacillus* species, group I-IV represented isolates that resembled *Geobacillus* species, group V represented isolates that resembled *Parageobacillus* species, and group XII and VI had isolates resembling *Paenibacillus, and Aeribacillus*, respectively. As with SDLC samples, in terms of the similarity scores between the isolates’ 16S rRNA genes, the *Geobacillus* isolates from SURF also had the high percentage identity of 96.01–99.86% and, here also, the *Parageobacillus* strain was grouped well close to the *Geobacillus* group with 96.09–98.51% identity between it and rest of the *Geobacillus* isolates. Within the *Geobacillus* isolates, the isolates belonged to species *thermodenitrificans, stearothermophilus, subterraneus*, and *thermoleovorans.* While the SURF *G*. *subterraneus* member (SURF-3S) had >97% identity to members of *G. thermodenitrificans,* the isolate resembling *G. stearothermophilus* had 99% identity to *G. thermoleovorans.* Based on this analysis, we believe that *Geobacillus* isolates in SURF samples had representatives from clade *G. thermodenitrificans,* as well as *G. thermoleovorans.* This observation fits well with the fact that members of *G. thermoleovorans* are known more for fermentation in micro-aerobic conditions with no shaking and aeration, and the low oxygen conditions in the SURF site are probably responsible for their presence in this site rather than the SDLC site, which is comparatively aerobic.

Within the isolates representing *Bacillus* (groups VII-X), as in the case of *Geobacillus*, the percentage similarity between the isolates within each group was, on average, again was high at 99.92%, 97.13%, 97.52%, and 98.54%, respectively. Nevertheless, within the group XI, represented by *Ureibacillus thermosphaericus* and *Bacillus kokeshiiformis*, the percentage similarity was just 89.65 despite these two species being grouped. Moreover, the isolate resembling *Bacillus kokeshiiformis* had only 89.31–91.41% identity with the other *Bacillus* groups altogether. A similar observation was seen while analyzing the positioning of isolates resembling *Bacillus smithii*, which had 90.48–92.94% identity with the rest of the *Bacillus* members.

The SURF sample also had isolates belonging to *Parageobacillus* (group V), and *Aeribacillus* (group VI), with an intra-cluster identity of 90.28–92.87% and 95.23%, that formed groups between the *Bacillus* and *Geobacillus* groups. Both the groups *Parageobacillus* and *Aeribacillus* shared only 89.11–91.16% and 88.21–92.15% identity percentages with *Bacillus* and *Geobacillus* groups. This validates the classification of *Parageobacillu,* and *Aeribacillus* as a separate genus. On the other hand, three isolates belonging to genus *Paenibacillus* (SURF-11S, 7S, 16S) appeared far from *Geobacillus*, and this also qualifies *Paenibacillus* as a separate genus, descending away from *Bacillus* in the phylogenetic tree.

Overall, according to phylogenetic analysis of the 16S rRNA gene sequences from both SDLC and SURF isolates, while the level of intraspecific similarity between the sequences from *Bacillus, Parageobacillus, Aeribacillus*, and *Paenibacillus* groups was 89.65–99.54%, 89.11–91.16%, 88.21–92.15%, and 90.28–92.87%, respectively; the degree of interspecific similarity between the sequences from *Geobacillus* isolates was found to be high (95.11–100%); and this identity in the cases of sequences within the individual *Geobacillus* groups representing a particular species was extremely high (98.8–99.9%). These findings indicate that 16S rDNA sequences alone could not resolve the phylogenetic relationships between the *Geobacillus* species used. Although the low phylogenetic power at the species level and weak discriminatory power of 16S rRNA for *Bacillus* genera have been reported in other studies too; in this study, the high variation between the *Bacillus members* was only found if the species were identified as either *B. smitthii, B. pumilus,* or *B. kokeshiiformi.* The rest of the isolates belonging to genus *Bacillus* were still found to have a similarity score of 97.01–99.02% between them, which again did not permit reliable species differentiation.

Indeed, the bootstrap value at the nodes of a few clusters obtained was too low to induce much confidence. This again suggests that 16S rDNA sequences alone were not able to resolve the phylogenetic relationships between the Firmicutes isolated in this study, and it may be difficult in general to distinguish strains of the same genus with 16S rDNA information. The 16S rDNA sequence comparison might be effective for the analysis of genera and higher orders. The gyrB sequence comparison, on the other hand, may better for defining the phylogenetic relationships at the species level. Therefore, in this study, we also performed gyrB identification on the isolates, which has a more variable nucleotide sequence.

### 3.3. Identification of the Isolates Using gyrB rRNA Marker

We performed the identification analysis by gyrB with the same isolates, to check if more differentiation can be obtained for the strains. As done for 16S analysis, we first grouped the isolates based on results of gyrB, and we found that, where 16S analysis gave a total of 29 differentiated isolates from 77 isolates from LC samples, gyrB analysis gave 30 individual isolates (Supplementary Table S3). However, with gyrB, more clarity was present in the groups formed within the phylogenetic tree.

#### 3.3.1. Analysis of gyR by Phylogenetic Tree and Multiple Sequence Alignment for SDLC Isolates

**Figure 7:**
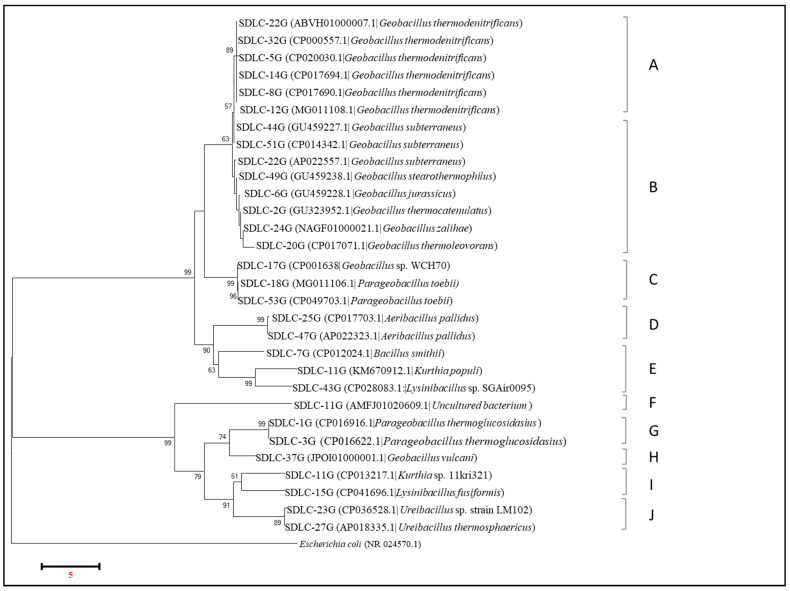
Neighbor-joining phylogenetic tree obtained from gyrB rRNA gene sequences of the isolates from the SDLC site. Sequences from this study are shown and isolates closest match from the NCBI database are given in parentheses. The scale bar represents the estimated sequence divergence with an estimated 5000 base substitutions per 1000 nucleotide positions. Percentages refer to significant bootstrap values of 1000 calculated trees. *E. coli* was used an outgroup.

Figure 7 below shows the phylogenetic tree that was obtained by aligning all the common isolates from the LC samples as identified by gyrB sequencing. Out of 77 isolates, 30 different strains have been identified, and these 30 isolates were found to form ten different groups (A–J) on a phylogenetic tree. Here, while group A consisted of all the *Geobacillus thermodenitrificans* strains, and group C consisted of strains identified to be *Parageobacillus tobeii*, including *Geobacillus* sp. WCH70, Group B was an assortment of *Geobacillus* strains identified to belong to many different species, i.e., *G. subtterraneus, G. stereothermophilus, G. jurassicus, G. thermocatenulatus, G. zalihae*, and *G. thermooleoverans.* Here, an interesting grouping was seen for an isolate identified as *G. vulcani* (group H) that was grouped with members of *Parageobacillus* as a sister group. Overall, while within group A the similarity score stood between 97.23–99.95%, the similarity score within the isolates in group B was variable. While there was 99.92–99.85% identity between the three *Geobacillus subterraneus* isolates, the identity score was 85.31–86.96% when the isolates belonged to a different *Geobacillus* species. Furthermore, the similarity score between the isolates from *G. thermodenitrificans* (group A) and isolates from group B (*G. subterraneus, G. tearothermophilus, G. jurassicus, G. thermocatenulatus, G. zalihae,* and *G. thermoleovorans)* was on an average between 90.59–85.83%. Consequently, it appears that gyrB is good enough to differentiate between different *Geobacillus* species, whereas 16S rRNA failed to do so, as evident in the similarity scores being as high as 95.11–100%.

Furthermore, with gyrB sequencing, the genus *Parageobacillus* occurred separately from the clade *Geobacillus*, which just had an average 42–77% match with members of *Geobacillus* and qualified to be a separate genus. In fact, members of *Parageobacillus* occurred in two different groups in the phylogenetic tree (Group C and G) with identity between the groups of only 40.17% and represented by ideally different species, i.e., *Parageobacillus toebii* (in Group C) and *Parageobacillus thermoglucosidans* (in group G). Moreover, while group C had 76.92–77.19% identity across *Geobacillus* species represented in group A, group G shared only 41.68–43.17% identity with *Geobacillus* species represented in group B. Hence, with gyrB, strains previously identified as *Geobacillus toebii* and *G. thermoglucosidans* by 16S rRNA were identified as infact *Parageobacillus toebii*, and *Parageobacillus thermoglucosidans.* And gyrB again appears to be good enough to differentiate isolates of *Geobacillus* from those of Parageobacillus, where 16S rRNA again failed to do so, giving the identity between the two genera of 98.70–99.44%.

In the phylogenetic tree, identification of some of the *Bacillus* members as belonging to genus *Kurthia* and *Lysinibacillus* was another interesting feature obtained with gyrB sequencing. Within Group E, the isolates identified as *Bacillus smithii*, *Kurthia populi*, and *Lysinibacillus* sp. occurred together with a similarity score within the group of 69.48–77.38%. Intriguingly, the isolates of *Kurthia* and *Lysinibacillus also* occurred in two different groupings in the phylogenetic tree (groups E and I) with a similarity score of approximately 77.38–77.91% between *Kurthia* and *Lysinibacillus* in both the groups and 40–41.3% between the two groups. While group E isolates of *Kurthia* and *Lysinibacillus* existed as a sister group with the isolates identified to belong to genus *Aeribacillus* (group D); Group I isolate of *Kurthia populli* existed closer to *Ureibacillus* (Group J), with identity between groups of *Aeribacillus* and *Ureibacillus* of just 38–39%. Indeed, within Group J, the two *Ureibacillus* isolates, despite belonging to the same genus but different species, shared 86.71% similarity. Here again, gyrB proved to be capable of differentiating at the species level within the *Ureibacillus, Bacillus, Kurthia,* and *Lysinibacillus* genera.

#### 3.3.2. Analysis of gyrB by Phylogenetic Tree and Multiple Sequence Alignment for SURF Isolates

**Figure 8:**
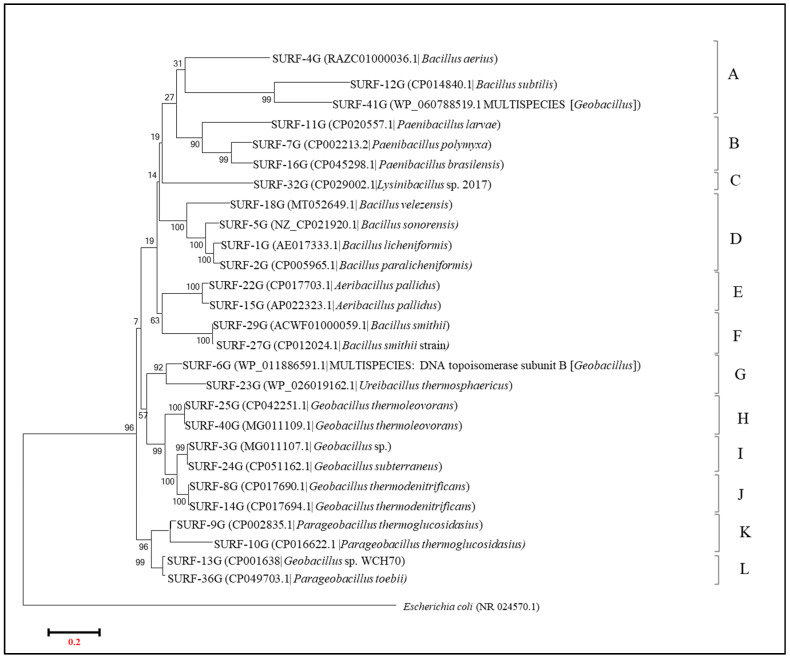
Neighbor-joining phylogenetic tree obtained from gyrB rRNA gene sequences of the isolates from the SURF site. Sequences from this study are shown, and isolates closest match from the NCBI database are given in parentheses. The scale bar represents the estimated sequence divergence with an estimated 200 base substitutions per 1000 nucleotide positions. Percentages refer to significant bootstrap values of 1000 calculated trees. *E. coli* was used as an out group.

Figure 8 below shows the phylogenetic tree that was obtained by aligning all the isolates (Supplementary Table S4) from the SURF samples as identified by gyrB sequencing. Here, out of 44 isolates, a total of 27 different strains have been identified. These 27 isolates were found to form twelve different groups (A–L) on a phylogenetic tree, hence depicting more heterogeneity in the isolates obtained from the SURF samples than the SDLC samples and also in comparison to 16S profiling obtained for the same SURF isolates which just had nine groups. The isolates belonging to genus *Geobacillus* were found to be divided into three groups H, I, and J, represented by characteristic species of *G. thermoleoverans*, *G. subterraneus*, and *G. thermodenitrificans.* Within these three *Geobacillus* groups, while the intraspecies percentage similarity of gyrase B identification within each group was 98.99–100%, the interspecies percentage identity amongst the *Geobacillus* groups was 85.80–91.37%. The exception to this grouping was the *Geobacillus* sp. strain WCH70 that, as in the earlier listing of gyrB from LC samples, was in the grouping with isolates of *Parageobacillus toebii* in group K. Overall in the SURF samples, there was a total of 2 distinct types of *Parageobacillus,* i.e., *P. thermoglucosidasius* (group K), and *P. toebii* (group L), with percentage similarity between two distinct species, and hence in between K and L group of 42.24–43.26%, and intraspecies similarity of 89.11%, and 89.13% within group K, and within group M, respectively. A similar kind of index was present within the *Paenibacillus* (group B), and *Aeribacillus* (group E) isolates where the strains, despite belonging to the same genus but different species, shared 76.06%, 70.54–88.48%, and 96.11% similarity between them, respectively.

Unlike in SDLC samples where most of the *Bacillus* species were identified as belonging to genus *Kurthia* or *Lysinibacillus*, the *Bacillus* isolates in SURF samples more or less maintained their species as identified by 16S, except for *Bacillus kokeshiiformis* that was still recognized as belonging to *Lysinibacter* genus and it formed a separate grouping (group C) to that of other *Bacillus* isolates. The *Bacillus* isolates overall in the SURF isolates belonged to many different species like *B. aerius, B. subtilis, B. velezensis, B. sonorensis, B. licheniformis, B. paralicheniformis*, and *B. smitthi*, where the isolates belonging to different *Bacillus* species shared identity between them in the range 53.38–96.08%. This again indicates the versatility of gyrase B as a gene marker to differentiate between the species of *Geobacillus, Parageobacillus, Paenibacillus, Aeribacillus,* and *Bacillus* more precisely compared to 16S rRNA analysis.

One notable feature is that, while performing identification using gyrB, some of the isolates from SURF were showing results as multispecies DNA topoisomerase IV of *Geobacillus* and DNA topoisomerase IV of *Ureibacillus,* with no particular species. This may be due to gyrB being a less explored identification marker so far for the members of Firmicutes. In this work, the gyrB primers used were designed based on previous work by Tuotora et al. (2010) and was more specific for Firmicutes isolates.

Since the phylum Firmicutes members are endospore-forming aerobic or facultatively anaerobic bacteria, that makes its species hardy in potentially harsh conditions. This property makes this phylum a phenotypically and phylogenetically diverse taxon and allows these bacteria to be distributed into most of the habitats on Earth. However, due to the endospores themselves, the Firmicutes members are known to be resilient to many traditional methods of DNA isolation and thus potentially go undetected and underrepresented in metagenomic diversity surveys [65,66,67]. Fillipidou et al., in their work in 2015, have highlighted this bias of metagenomic studies, with only a minor fraction of the community assigned to Firmicutes, while they evaluated the representation of endospore-forming Firmicutes in 73 published metagenomic datasets [67]. Counter to this usual bias, we found Firmicutes to be the most dominant phylum of microbial life in both SDLC and SURF environments. Similarly, Actinobacteria (many of which have a spore stage) was detected as the second most dominant phylum in the SDLC sample, at 5.5%. We believe that the metagenomic analyses showing positive hits for the Firmicutes 16S rRNA gene in both of the two sites studied are due to the DNA extraction method we applied, and/or to the depth of sequencing that is hard to establish. The earliest and highly vital step in any metagenomic project is the extraction of DNA, which should be representative of all cells in the sample and involve a sufficient quality and amount for subsequent sequencing. The MINES method which makes use of a gentle preprocessing technique based on bead beating, combined with a multi-lytic polyzyme treatment, has been shown to be suitable enough for unbiased extraction of total genomic DNA from environmental samples for functional metagenomic studies [43]. Hence, we consider that the “MINES” DNA isolation method used in this study [43], along with the use of 16S rRNA metagenomic sequencing, facilitates the recovery of DNA representative of endospore-forming communities from the environmental samples, without any evident bias.

To further expand our understanding of the distribution of Firmicutes, a culture-dependent approach was applied to samples collected from the SDLC and SURF sites. This investigation resulted in the isolation of 77 isolates from SDLC samples, and 40 isolates from SURF samples, spanning five families (*Geobacillus*, *Bacillus*, *Aeribacillus, Paenibacillus, Parageobacillus*) and also included a representative of Uncultured *Thermoactinomyces.* Phylogenetic analysis of these isolates based on 16S rRNA gene sequence data indicated that isolates from both the samples were predominantly the strains belonging to genus *Geobacillus*, representing 72.4% and 41.3% of the total isolates from the SDLC and SURF samples, respectively. It has been proven that *Geobacillus* have a worldwide distribution in environments spanning hot springs to even cold climates, and this is almost certainly, in substantial portion, due to the adaptive features of their spores, which enable them to lie quiescent and achieve high population densities after accumulating gradually over an elongated timeline. The isolation of *Geobacillus* representatives from the host compost (SDLC) at 60° and SURF sediments at 45 °C in high percentage, after a preconditioned enrichment, points to the excellent capability of this genus to colonize niches with varying temperatures, and low availability of nutritional compounds, due to their generally simple nutritional needs. The Black Hills region of South Dakota has never been studied earlier for the bacterial diversity of Firmicutes and its taxa. Hence, the isolation of strains of this phylum and genus *Geobacillus*, found in high number in this study, should significantly add to the knowledge of the cultivation based microbial diversity of endospore formers. It is commonly understood that the culture-independent methods reveal more diversity of microbial communities in soil than culture-dependent methods. However, in this study, more *Geobacillus and Bacillus* like bacterial genera were detected by the culture-dependent method rather than the culture independent metagenomic approach. In the first place, this discrepancy can be because of the specific enrichment procedure that was performed preceding the culture isolations for aerobic endospore forming Firmicutes members in this study. However, other researchers have also recently detected a higher percentage of cultivable *Bacillus*-like genera from soils [1,68,69]. Together, these findings indicate that DNA based metagenomic sequencing studies may often miss some information from some categories of Firmicutes genera that are present in the sites, but at low abundance. However, the explanation for these finding should be a matter of further research.

## 4. Summary

In brief, the results showed differences in microbial composition and community structure in the two locations studied. With the total bacterial OTUs of 18,371 and 80,885, the most dominant phylum in both locations was Firmicutes composing 86.1% (15,814 OTU’s) and 69.6% (60,002 OUTs) in the South Dakota Landfill Compost (SDLC) and Sanford Underground Research Facility (SURF) sites, respectively. At the class level, the most dominant class in the SDLC was Bacilli at 83.2%, followed by Clostridia at 2.9%. In the SURF sediments, by contrast, there was a dominance of Clostridia at 45.8% over Bacilli at 20.1%. This observation may arise from the presence of comparatively more oxygen in the SDLC samples. Since Bacilli members are facultative anaerobes, with clostridia preferring anaerobic conditions, this might have favored more Bacilli in SDLC samples than in SURF sediments. Also, as revealed by a Shannon–Weaver index of 3.03 in the SDLC samples vs. 1.09 in the SURF samples, the bacterial diversity at levels of lower taxa was apparently higher in the SDLC site over SURF, which can be attributed to higher nutrient content in the compost samples affecting bacterial growth and inducing the richness and diversity of the bacterial community. Hence, this study provides an understanding of the microbial community structure in the Black Hills, South Dakota, USA, and an insight into the ubiquitous presence of endospore forming Firmicutes in both the sites investigated. Members of the Firmicutes are present in landfill sites with suggestive role in landfill cellulose decomposition. However, finding a population of Firmicutes in high abundance in the SURF site was surprising, and this suggests that Firmicutes can facilitate bioremediation of sites contaminated with metals.

We also analyzed the genotypic and phenotypic diversity and phylogenetic relationships among the 29 differential Firmicutes isolate from SDLC and 29 of the isolates from SURF using 16S rRNA and gyrB as the identification markers. The results indicated that while both the markers are sufficient for bacterial identification, taxonomic analysis, and monitoring of bacteria in the natural environment, gyrB sequence comparison appeared more useful than 16S rDNA information for distinguishing between closely related strains of the same genus while offering sufficient confidence to guarantee species identity.

## Figures and Tables

**Table 1:** Physio-chemical properties of the samples used in this study.

Sample	50:50-Yard Waste/MSW Compost Pile (SDLC)	Sediments (SURF)
Sampling Site	SDLC, SD	SURF, SD
Temperature (°C)	60 ± 5.0	40 ± 3.0
pH (units)	5.32 ± 0.3	6.40 ± 0.2
Moisture (%)	25 ± 0.3	14 ± 0.2
Dissolved oxygen (DO) (ppm)	3.5 ± 0.3	3.1 ± 0.3
Organic matter (% dry weight)	75.0 ± 0.3	24.0 ± 0.3
Organic Carbon (% dry weight)	37.0 ± 3.20	5.1 ± 0.67
Total Nitrogen (%)	1.40 ± 0.02	1.57± 0.08
C/N ratio	27 ± 0.02	8.0 ± 0.03
Ammonia (mg/Kg)	42	ND

MSW: Municipal solid waste; SDLC: South Dakota Landfill Compost; SURF: Sanford Underground research Facility; SD: South Dakota, USA; C/N: Carbon: Nitrogen; ND: Not determined.

**Table 2:** Results of 16S rRNA amplicon sequencing for SDLC and SURF at the phylum level. The relative abundance of bacterial classes in phyla with >1% representation in either of the samples is also included.

Phylum	% Phylum in SURF Sediments (OTU)	% Phylum in SDLC Compost (OTU)
Acidobacteria	0.65 (557)	0 (0)
Actinobacteria Actinobacteria	0.03 (20) 0.03	5.47 (1005) 5.47
Aquificae	0.03 (22)	0 (0)
Bacteroidetes Bacteroidia Cytophagia Sphingobacteriia Unclassified	1.89 (1717) 1.0 0 0 0.89	0.56 (103) 0 0.06 0.5 0
Candidatus Atribacteria	0.01 (10)	0 (0)
Chloroflexi	0.88 (756)	0 (0)
Deferribacteres	0 (0)	0 (0)
Deinococcus-Thermus	0 (0)	0.10 (25)
Firmicutes Bacilli Clostridia Negativicutes Tissierellia Unclassified	69.60 (60,002) 20.10 45.80 0.10 0.10 3.50	86.10 (15,814) 83.20 2.90 0 0 0
Lentisphaerae	0 (0)	0 (0)
Planctomycetes	0.37 (322)	0 (0)
Proteobacteria α-proteobacteria β-proteobacteria δ-proteobacteria ϒ-proteobacteria ε-proteobacteria	5.44 (2998) 4.10 1.14 0.20 0 0	6.63 (1216) 0.33 0.80 0 5.46 0
Spirochaetes	0.44 (377)	0 (0)
Synergistetes	0 (0)	0 (0)
Tenericutes	0 (0)	0 (0)
Unclassified	16.34 (14,104)	0.53 (97)
No Hit	4.32 (4335)	0.61 (111)

## Data Availability

The data presented in this study are openly available in the U.S. National Center for Biotechnology Information and is available through accession number PRJNA684582.

## Supplementary Materials

The following are available online at https://www.mdpi.com/2076-2607/9/1/113/s1, Table S1: Isolates (growth temperature 60 °C) from SDLC Site, identified using 16S rRNA sequencing; Table S2: Isolates (growth temperature 45 °C) from SURF Site, identified using 16S rRNA sequencing; Table S3: Isolates (growth temperature 60 °C) from SDLC Site, identified using gyrB rRNA sequencing; Table S4: Isolates from SURF Site, identified using gyrB sequencing.
