# Supplementary material for: Metagenomics and Culture Dependent Insights into the Distribution of Firmicutes across Two Different Sample Types Located in the Black Hills Region of South Dakota, USA
Source: Microorganisms. 2021 Jan 6;9(1):113. doi: 10.3390/microorganisms9010113 (PMC7825136; doi:10.3390/microorganisms9010113)
Supplement: Supplementary file 1 [file microorganisms-09-00113-s001.pdf]

Supplementary Tables

Supplementary Table S1. Isolates (growth temperature 60°C) from SDLC Site, identified using 16S rRNA sequencing.

	SDLC isolate	Representative isolate	Accession number	Sequence length (bp)	Identity found in NCBI BLAST search[b]	Sequence identity (%)
1	LC 1	SDLC-1S	MW284848	1424	HQ703944.1|*Geobacillus* sp. E263	99.29
2	LC 2	SDLC-2S	MW284849	1320	LT745877.1| *Geobacillus* sp. 3BC	100.00
3	LC 3	SDLC-3S	MW284850	1357	HM989019.1| *Geobacillus* sp. TS3-9	99.64
4	LC 4, 9,10,32	SDLC-4S	MW284851	1461	LC379068.1|*Aneurinibacillus migulanus*	100.00
5	LC 5	SDLC-5S	MW284852	1347	MT634740.1|*Geobacillus thermodenitrificans*	98.45
6	LC 6, 17	SDLC-6S	MW284853	1440	MN252921.1|*Geobacillus thermodenitrificans*	99.86
7	LC 7	SDLC-7S	MW284854	1487	KJ567099.2| *Bacillus* sp.	99.98
8	LC 8, 14, 29, 38, 56, 71	SDLC-8S	MW284855	1376	MF965060.1|*Geobacillus thermodenitrificans*	99.41
9	LC 11, 30, 35	SDLC-11S	MW284856	1468	EU685818.1| *Bacillus* sp. PK9	92.36
10	LC 12, 36, 41	SDLC-12S	MW284857	1459	KT148963.1|*Geobacillus thermodenitrificans*	99.73
11	LC 13, 22, 46, 52, 55, 59,60,62	SDLC-13S	MW284935	1433	AM179887|*Bacillus* sp. C81	98.80
12	LC 15,19, 42, 43, 58	SDLC-15S	MW284858	1454	NR_133975.1|*Bacillus kokeshiiformis*	100.00
13	LC 16, 45	SDLC-16S	MW284859	1397	MN822300.1| *Bacillus pumilus*	100.00
14	LC 18	SDLC-18S	MW284860	1431	MF040267.1 | *Parageobacillus toebii*	100.00
15	LC 20, 34, 57, 74	SDLC-20S	MW284861	1399	NR_114014.1| *Geobacillus zalihae*	99.69
16	LC 21	SDLC-21S	MW284862	1452	CP038860.1| *Geobacillus kaustophilus*	98.02
17	LC 23, 24	SDLC-23S	MW284863	1409	DQ401072|*Anoxybacillus kualawohkensis*	91.20
18	LC 25, 26, 47	SDLC-25S	MW284864	1443	NR_026515.1|*Aeribacillus pallidus*	100.00
19	LC 27, 61, 64	SDL-27S	MW284865	1399	NR_040961.1|*Ureibacillus thermosphaericus*	99.86
20	LC 28	SDLC-28S	MW284866	1406	NR_132400.1|*Geobacillus subterraneus*	97.95
21	LC 31	SDLC-31S	MW284934	1418	NR_041761.1| *Thermoactinomyces vulgaris*	95.38

22	LC 33	SDLC-33S	MW284867	1301	GQ293457.1 | *Bacillus smithii*	96.90
23	LC 37,40	SDLC-37S	MW284868	1490	CP042251.1 | *Geobacillus thermoleovorans*	97.39
24	LC 39, 48, 50, 65, 70	SDLC-39S	MW284869	1478	AY608975.1 | *Geobacillus kaue*	99.78
25	LC 44	SDLC-44S	MW284870	1436	AF276307.1 | *Geobacillus subterraneus*	99.86
26	LC 49	SDLC-49S	MW284871	1399	MN519400.1 | *Geobacillus stearothermophilus*	99.86
27	LC 51, 73	SDLC-51S	MW284872	1410	NR_025109.1 | *Geobacillus subterraneus*	96.36
28	LC 53, 54	SDLC-53S	MW284873	1437	NR_116984.1 | *Geobacillus toebii*	98.27
29	LC 63, 66,67,68,69, 72, 75,76,77	SDLC-63S	MW284874	1399	NR_108200.1 | *Geobacillus thermodenitrificans*	93.93

[b] Sequence identity shared between the representative isolate and its nearest type strain

Supplementary Table S2. Isolates (growth temperature 45°C) from SURF Site, identified using 16S rRNA sequencing.

	SURF isolate	Representative isolate	Accession number	Sequence length (bp)	Identity found in NCBI BLAST search[b]	Sequence identity (%)
1	SURF 1	SURF 1S	MW282852	1482	CP033198.1 | *Bacillus paralicheniformis*	97.25
2	SURF 3, 24	SURF 3S	MW282853	1438	NR_132400.1 | *Geobacillus subterraneus*	99.86
3	SURF 4	SURF 4S	MW282854	1460	NR_042338.1 | *Bacillus aerius*	97.12
4	SURF 5	SURF 5S	MW282855	1456	NR_113993.1 | *Bacillus sonorensis*	96.02
5	SURF 6, 31, 34, 35	SURF 6S	MW282856	1503	CP017690.1 | *Geobacillus thermodenitrificans*	99.93
6	SURF 7	SURF 7S	MW282857	1383	KY859838.1 | *Paenibacillus polymyxa*	99.86
7	SURF 8, 26	SURF 8S	MW282858	1524	JN647924.1 | *Geobacillus stearothermophilus*	97.79
8	SURF 9	SURF 9S	MW282859	1558	NR_043022.2 | *Parageobacillus thermoglucosidans*	100.00
9	SURF 10	SURF 10S	MW282860	1481	NR_116983.1 | *Parageobacillus thermoglucosidasius*	95.92
10	SURF 11	SURF 11S	MW282861	1490	CP019652.1 | *Paenibacillus larvae*	99.80
11	SURF 12	SURF 12S	MW282862	1424	NR_113265.1 | *Bacillus subtilis*	96.10
12	SURF 13	SURF 13S	MW282863	1476	CP001638.1 | *Geobacillus* sp.	100.00
13	SURF 14, 28	SURF 14S	MW282864	1429	AB546234.1 | *Geobacillus thermodenitrificans*	99.93
14	SURF 15	SURF 15S	MW282865	1462	KJ842637.1 | *Aeribacillus pallidus*	100.00
15	SURF 16,17	SURF 16S	MW282866	1514	CP001793.1 | *Paenibacillus* sp.	97.85
16	SURF 18	SURF 18S	MW282867	1494	CP054714.1 | *Bacillus velezensis*	97.88
17	SURF 2, 19,20,21	SURF 2S	MW282868	1425	MT642946.1 | *Bacillus licheniformis*	95.89
18	SURF 22	SURF 22S	MW282869	1459	NR_026515.1 | *Aeribacillus pallidus*	99.87
19	SURF 23	SURF 23S	MW282870	1391	NR_040961.1 | *Ureibacillus thermosphaericus*	100.00
20	SURF 25, 30, 40	SURF 25S	MW282871	1491	NR_115286.2 | *Geobacillus thermoleovorans*	100.00
21	SURF 27	SURF 27S	MW282872	1504	CP012024.1 | *Bacillus smithii*	96.78
22	SURF 29	SURF 29S	MW282873	1431	GQ293457.1 | *Bacillus smithii*	99.86
23	SURF 32	SURF 32S	MW282874	1454	NR_133975.1 | *Bacillus kokeshiiformis*	99.87
24	SURF 33, 37, 38, 39	SURF 33S	MW282875	1377	MN252917.1 | *Geobacillus thermodenitrificans*	95.85

25	SURF 36	SURF 36S	MW282876	1468	NR_116984.1 | *Parageobacillus toebii*	96.92
26	SURF 41	SURF 41S	MW282877	1357	MN830248.1 | *Geobacillus thermoleovorans*	97.55

[b] Sequence identity shared between the representative isolate and its nearest type strain

Supplementary Table S3. Isolates (growth temperature 60°C) from SDLC Site, identified using gyrB rRNA sequencing.

	SDLC Isolate	Representative isolates	Sequence length (bp)	Identity found in NCBI BLAST search	% identity
1	LC 1	SDLC-1G	1923	CP016916.1|*Parageobacillus thermoglucosidasius*	100
2	LC 2	SDLC-2G	1864	GU323952.1 |*Geobacillus thermocatenulatus*	99.98
3	LC 3	SDLC-3G	1924	CP016622.1|*Parageobacillus thermoglucosidasius*	80.82
4	LC 4, 9,10,41, 46,55, 59, 60	SDLC-4G	1923	CP017694.1| *Geobacillus thermodenitrificans*	99.70
5	LC 5, 8, 61, 62, 70	SDLC-5G	1920	CP020030.1|*Geobacillus thermodenitrificans*	85.00
6	LC 6	SDLC-6G	1178	GU459228.1 |*Geobacillus jurassicus*	99.60
7	LC 7,33,	SDLC-7G	1400	CP012024.1| *Bacillus smithii*	100.00
8	LC11,30, 35	SDLC-11G	821	KM670912.1| *Kurthia populi*	95
9	LC 12, 13, 38	SDLC-12G	1920	MG011108.1| *Geobacillus thermodenitrificans*	81.44
10	LC14,29,32,48,50,56,63,	SDLC-14G	1920	CP017690.1|*Geobacillus thermodenitrificans*	99.1
11	LC 15, 19,42, 58	SDLC-15G	1929	CP041696.1|*Lysinibacillus fusiformis*	100.00
12	LC 43	SDLC-43G	1744	CP028083.1:|*Lysinibacillus* sp. SGAir0095	100.00
13	LC 16, 45	SDLC-16G	1431	CP013217.1| *Kurthia* sp. 11kri321	80.93
14	LC 17,21	SDLC-17G	1818	CP001638| *Geobacillus* sp. WCH70	99.60
15	LC 18	SDLC-18G	1706	MG011106.1| *Parageobacillus toebii*	99.70
16	LC 20	SDLC-20G	1573	CP017071.1|*Geobacillus thermoleovorans*	98.70
17	LC 22, 52	SDLC-22G	1923	ABVH01000007.1| *Geobacillus thermodenitrificans*	99.84
18	LC 23,24,	SDLC-23G	1923	CP036528.1| *Ureibacillus* sp. strain LM102	83.13
19	LC 25,26	SDLC-25G	1923	CP017703.1| *Aeribacillus pallidus*	82.43
20	LC 27,64	SDLC-27G	1881	AP018335.1| *Ureibacillus thermosphaericus*	99.79
21	LC 28	SDLC-28G	1881	AP022557.1| *Geobacillus subterraneus*	99.99
22	LC 31	SDLC-31G	1321	AMFJ01020609.1| *Uncultured bacterium*	97.00
23	LC 34, 57,74	SDLC-34G	1923	NAGF01000021.1| *Geobacillus zalihae*	99.56
24	LC 36, 39,40, 65,66,67,68,75,76,77	SDLC-36G	1892	CP000557.1| *Geobacillus thermodenitrificans*	99.87
25	LC 37	SDLC-37G	1928	JPOI01000001.1| *Geobacillus vulcani*	98.43
26	LC 44	SDLC-44G	1181	GU459227.1| *Geobacillus subterraneus*	100.00
27	LC 47,	SDLC-47G	1932	AP022323.1| *Aeribacillus pallidus*	98.25
28	LC 49	SDLC-49G	1158	GU459228.1	100.00
29	LC 51, 73	SDLC-51G	1820	CP014342.1| *Geobacillus subterraneus*	100.00
30	LC 53, 54	SDLC-53G	1711	CP049703.1| *Parageobacillus toebii*	100.00

Supplementary Table S4. Isolates from SURF Site, identified using gyrB sequencing.

	SURF Isolate	Representative isolate	Sequence length	Identity found in NCBI BLAST search	Sequence identity (%)
1	SURF 1	SURF-1G	1911	AE017333.1| *Bacillus licheniformis*	79.69
2	SURF 2, 19	SURF-2G	1922	CP005965.1| *Bacillus paralicheniformis*	99.28
3	SURF 3	SURF-3G	1917	MG011107.1| *Geobacillus* sp.	79.39
4	SURF 4	SURF-4G	1968	RAZC01000036.1| *Bacillus aerius*	90.20
5	SURF 5	SURF-5G	1885	NZ_CP021920.1| *Bacillus sonorensis*	100.00
6	SURF 6, 31,35	SURF-6G	1920	WP_011886591.1| MULTISPECIES: DNA topoisomerase subunit B [*Geobacillus*]	81.41
7	SURF 7, 17	SURF-7G	1907	CP002213.2| *Paenibacillus polymyxa*	79.80
8	SURF 8, 26,33,34	SURF-8G	1920	CP017690.1| *Geobacillus thermodenitrificans*	81.44
9	SURF 9,	SURF-9G	1920	CP002835.1| *Parageobacillus thermoglucosidasius*	100.00
10	SURF 10	SURF-10G	1920	CP016622.1 |*Parageobacillus thermoglucosidasius*	82.94
11	SURF 11	SURF-11G	1908	CP020557.1| *Paenibacillus larvae*	77.44
12	SURF 12	SURF-12G	1968	CP014840.1| *Bacillus subtilis*	71.45
13	SURF 13	SURF-13G	1920	CP001638| *Geobacillus* sp. WCH70	81.45
14	SURF 14,28,37,38,39	SURF-14G	1923	CP017694.1| *Geobacillus thermodenitrificans*	100.00
15	SURF 15	SURF-15G	1926	AP022323.1| *Aeribacillus pallidus*	100.00
16	SURF 16	SURF-16G	1892	CP045298.1| *Paenibacillus brasilensis*	98.80
17	SURF 18, 20	SURF-18G	1914	MT052649.1| *Bacillus velezensis*	77.36
18	SURF 22	SURF-22G	1923	CP017703.1| *Aeribacillus pallidus*	78.25
19	SURF 23	SURF-23G	1923	WP_026019162.1| *Ureibacillus thermosphaericus*	99.23
20	SURF 24	SURF-24G	1923	CP051162.1| *Geobacillus subterraneus*	100.00
21	SURF 25, 30,	SURF-25G	1880	CP042251.1| *Geobacillus thermoleovorans*	99.10
22	SURF 27	SURF-27G	1884	CP012024.1| *Bacillus smithii* strain	99.00
23	SURF 29	SURF-29G	1932	ACWF01000059.1| *Bacillus smithii*	99.90
24	SURF 36	SURF-36G	1920	CP049703.1| *Parageobacillus toebii*	99.95
25	SURF 40	SURF-40G	1923	MG011109.1| *Geobacillus thermoleovorans*	100.00
26	SURF 41	SURF-41G	1935	WP_060788519.1 MULTISPECIES [*Geobacillus*]	83.71
27	SURF 32	SURF-32G	1881	CP029002.1|*Lysinibacillus* sp. 2017	100.00
